# Challenges faced by the Medical Editors in COVID19 Pandemic era

**DOI:** 10.12669/pjms.36.5.3009

**Published:** 2020

**Authors:** Shaukat Ali Jawaid, Masood Jawaid

**Affiliations:** 1 Shaukat Ali Jawaid Chief Editor, Pakistan Journal of Medical Sciences Karachi - Pakistan; 2 Masood Jawaid Associate Editor, Pakistan Journal of Medical Sciences Karachi - Pakistan

The latest threat of COVID19 pandemic has jolted the whole world and affected every segment of the society, forced people to change their working style which is not so easy for everyone. Medical Editors were no exception and COVID19 pandemic has affected their working as well. While the leading biomedical journals of the world with huge staff and adequate resources were quick to shift to work from home, it was not possible or easy for many journals in the developing, countries. Firstly they did not have the resources to begin with, secondly the lockdowns were announced so suddenly and thirdly most of the staff were not trained to work from home. The result was that manuscript already submitted, could not be processed further in time and got delayed. The peer review process also got disrupted and the normal flow of working could not materialize.

The authors are most often most impatient and wish to get their manuscripts published as soon as possible that is why the authors are considered the most dangerous pressure groups which the editors have to face.^1^ Authors had more time due to lockdowns and they kept up their pressure making the life of the editors quite uncomfortable. As soon the lockdown were relaxed, the editors had too many manuscripts under process and pending and most of their time was spent on clearing this backlog. Some journals even started refusing new submissions for some time while others were more cautions and accepted a very few selected new submissions for further processing after editor’s triage, initial screening.

Since lockdowns provided ample time for the researchers and authors, they used this opportunity to complete their research projects, pending write-ups and accelerated their submissions. The result was an overwhelming increase in the new submissions with most of the peer reviewed biomedical journals. There were some positive developments as well linked to this COVID19 pandemic. There was sudden surge in research activities, numerous studies were planned, executed on COVID19 pandemic, many new drug trials are under way starting from finding a possible vaccine to testing efficacy of already available anti-viral preparations besides finding new effective cost effective drugs for its treatment. Numerous medical journals all over the world have published special supplements on COVID19 pandemic as hundreds of papers are getting published daily related to this virus. Easy availability of so much literature on this virus also encouraged the healthcare professionals to plan systemic reviews, meta-analysis and reviews. Some researchers have even started critically looking at the information available on COVID19 on YouTube, analyzing it for their quality and usefulness. Categorization of the submissions on COVID19 has also been a dilemma for the editors as in many cases, the authors did not like to accept the editor’s decisions. It also had something to do with the regulatory bodies of various countries which give different weightage to different categories of manuscripts. In fact almost everyone who has interest in medical writing is now interested to write a review or short communication on COVID19 and medical journals are getting innumerable requests to process them which of course is not possible.

When we decided to publish a special supplement devoted to COVID19, we selected a team of thirteen peer reviewers who were requested to review the manuscripts received within a week’s time at the most. The authors were also asked to respond to the reviewers comments immediately, sending the revised manuscripts immediately so as to accelerate the whole publication process. All those submissions before being sent for external review had their initial screening, got through the editor’s triage, similarity check using iThenticate as well as internal review to make the job of the reviewers easy. Hence, we were able to select and publish thirty three manuscripts in different categories from authors from Pakistan and overseas. The supplement was published in a record time of just seven weeks without compromising on quality and peer review.^2^

Since in this era of COVID19 pandemic, most of the teaching institutions, medical colleges and medical universities being no exception have also opted for online education, it has opened yet another area for research. Hence, editors are now getting many manuscripts related to online education as well. Though every institution is trying to opt for it but, the faculty is not trained to use the new technology to facilitate online education and it will take some time when the faculty and students will start enjoying these online education activities.

On the other hand, problems faced by the medical editor have multiplied as they are now receiving more and more manuscripts just related to different aspects of COVID19 with the result that manuscripts on other disorders, subjects do not get the same attention. The processing of routine submissions get delayed much to the agony of the authors who are keen to share their information with other colleagues soon. However, Peer Review process is time consuming and there is no quick fix solution. This has encouraged some authors to post their manuscripts on sites like *medRxiv*. It is sponsored by Yale University Open Data Access.^3^ It takes far less time and efforts while its impact is noted to be considerable. Many authors have found a new way to pressurize the editors by putting the word COVID19 in the title and then request the editors of accelerated peer review. However, if one starts compromising on peer review, then there will be no difference left in information carried by good quality peer reviewed medical journals and the information being made available through Social Media. Hence the Editors are supposed to uphold professional ethics, make the peer review process more rapid. However, it is also important that Peer Review maintains its highest possible standards.^4^

## References

[1] Jawaid SA (2004). Problems faced by editors of peer reviewed medical journals. Saudi Med J.

[2] COVID-19 Supplement 2020 Special Issue https://www.pjms.org.pk/index.php/pjms/issue/view/13.

[3] https://www.medrxiv.org/.

[4] Blog by Milton Packer.

